# Droplet digital PCR-based detection of circulating tumor DNA from pediatric high grade and diffuse midline glioma patients

**DOI:** 10.1093/noajnl/vdab013

**Published:** 2021-01-27

**Authors:** Elisa Izquierdo, Paula Proszek, Giulia Pericoli, Sara Temelso, Matthew Clarke, Diana M Carvalho, Alan Mackay, Lynley V Marshall, Fernando Carceller, Darren Hargrave, Birgitta Lannering, Zdenek Pavelka, Simon Bailey, Natacha Entz-Werle, Jacques Grill, Gilles Vassal, Daniel Rodriguez, Paul S Morgan, Tim Jaspan, Angela Mastronuzzi, Mara Vinci, Michael Hubank, Chris Jones

**Affiliations:** 1 Division of Molecular Pathology, Institute of Cancer Research, London, UK; 2 Molecular Diagnostics, Royal Marsden Hospital NHS Trust, Sutton, UK; 3 Department of Onco-haematology, Cell and Gene Therapy, Bambino Gesù Children’s Hospital-IRCCS, Rome, Italy; 4 Division of Clinical Studies, The Institute of Cancer Research, London, UK; 5 Children & Young People’s Unit, Royal Marsden Hospital NHS Trust, Sutton, UK; 6 Department of Haematology and Oncology, UCL Great Ormond Street Institute for Child Health, London, UK; 7 Department of Pediatrics, Institute of Clinical Sciences, Queen Silvia Children’s Hospital, University of Gothenburg, Gothenburg, Sweden; 8 Department of Pediatric Oncology, University Hospital Brno – Children’s Hospital, Brno, Czechia; 9 Department of Paediatric Oncology, Great North Children’s Hospital, Newcastle University Center for Cancer, Newcastle upon Tyne, UK; 10 Pediatric Onco-Hematology Department, University Hospital of Strasbourg, Strasbourg, France; 11 UMR CNRS 7021, Laboratory Bioimaging and Pathologies, Tumoral Signaling and Therapeutic Targets team, Faculty of Pharmacy, Illkirch, France; 12 Pediatric and Adolescent Oncology and INSERM Unit U981, Team Genomics and Oncogenesis of Pediatric Brain Tumors, Gustave Roussy and Paris Saclay University, Villejuif, France; 13 Medical Physics and Clinical Engineering, Nottingham University Hospital Trust Nottingham University Hospital Trust, Nottingham, UK; 14 Department of Radiology, Nottingham University Hospital Trust, Nottingham University Hospital Trust, Nottingham, UK

**Keywords:** cfDNA, ctDNA, plasma, CSF, DIPG, HGG

## Abstract

**Background:**

The use of liquid biopsy is of potential high importance for children with high grade (HGG) and diffuse midline gliomas (DMG), particularly where surgical procedures are limited, and invasive biopsy sampling not without risk. To date, however, the evidence that detection of cell-free DNA (cfDNA) or circulating tumor DNA (ctDNA) could provide useful information for these patients has been limited, or contradictory.

**Methods:**

We optimized droplet digital PCR (ddPCR) assays for the detection of common somatic mutations observed in pediatric HGG/DMG, and applied them to liquid biopsies from plasma, serum, cerebrospinal fluid (CSF), and cystic fluid collected from 32 patients.

**Results:**

Although detectable in all biomaterial types, ctDNA presented at significantly higher levels in CSF compared to plasma and/or serum. When applied to a cohort of 127 plasma specimens from 41 patients collected from 2011 to 2018 as part of a randomized clinical trial in pediatric non-brainstem HGG/DMG, ctDNA profiling by ddPCR was of limited use due to the small volumes (mean = 0.49 mL) available. In anecdotal cases where sufficient material was available, cfDNA concentration correlated with disease progression in two examples each of poor response in *H3F3A*_K27M-mutant DMG, and longer survival times in hemispheric *BRAF*_V600E-mutant cases.

**Conclusion:**

Tumor-specific DNA alterations are more readily detected in CSF than plasma. Although we demonstrate the potential of the approach to assessing tumor burden, our results highlight the necessity for adequate sample collection and approach to improve detection if plasma samples are to be used.

**Key Points**

- We show the utility of ddPCR techniques to reliably detect ctDNA of all major subtypes of pHGG/DMG from plasma, serum, cystic fluid, and CSF.
- We show that cfDNA can be used to track disease progression in both hemispheric and midline tumors.

**Importance of the Study**

Children with high-grade and diffuse midline glioma have an invariably fatal outcome, and with surgical resection impossible when occurring in the brainstem (diffuse intrinsic pontine glioma, DIPG), such non-invasive specimens have the potential to play a vital role in tumor diagnosis and disease monitoring. Here we show the utility of sensitive and specific ddPCR techniques to reliably detect circulating tumor DNA of all major subtypes of pHGG/DMG (including DIPG), from plasma, serum, cystic fluid, and CSF. We further screened very limited quantities of serial plasma samples collected as part of the HERBY clinical trial to show that cell-free DNA can be used to track progression in both hemispheric and midline tumors. These data provide a rationale for the incorporation of such liquid biopsy collection into future clinical trials for inclusion and molecular stratification, monitoring of treatment response, and for guiding novel therapeutic interventions at relapse.

The incorporation of tissue molecular profiling in patients with pediatric high-grade glioma (pHGG), diffuse intrinsic pontine glioma (DIPG) and other diffuse midline glioma (DMG) into clinical practice has been demonstrated to be essential to guide treatment decisions for these patients.^1,2^ However, this requires invasive neurosurgical procedures, which are frequently associated with a risk of morbidity or mortality.^3–5^ These risks are of particular concern for tumors located within the brainstem, such as DIPG, where biopsy is technically very challenging and is associated with a risk of significant complications.^3,4^

The study of liquid biopsy has emerged as an alternative and/or complementary approach to tumor biopsy. Liquid biopsy analysis is comprised of the study of tumor derived material from any biological fluids including blood, cerebrospinal fluid (CSF), urine, and saliva. In this context, cell-free DNA (cfDNA), extracted from different biofluid sources, is used to assess tumor-specific alterations in a less invasive manner. The fraction of cfDNA derived from tumor cells is known as circulating tumor DNA (ctDNA). A benefit of liquid biopsy analysis is the ability to correlate the presence of driver mutations with tumor burden and response to therapy at multiple time-points, avoiding the risks, costs, and need for the expertise of surgical intervention. In this context, many pediatric gliomas are characterized by hotspot driver mutations (H3.1/H3.3_K27M, H3.3_G34R/V, *BRAF*_V600E, *IDH1*_R132H)^6^ or by single fusion events (*ETV6*:*NTRK3, KIAA1549:BRAF*).^7,8^ This makes them perfect candidates for the use of ctDNA to monitor treatment response enabling early detection of tumor progression over the course of the disease.

The presence of ctDNA from plasma has been demonstrated in a range of pediatric solid tumors.^9–14^ Despite concerns regarding the utility of such approaches in brain tumors,^15^ several studies have illustrated that ctDNA can be detected in CSF from a variety of pediatric and adult central nervous system (CNS) malignancies.^16–20^ In particular, Wang and colleagues found molecular alterations in 74% of patients from ctDNA derived from CSF, obtaining an average of 417 ng of ctDNA in an average of 4.8 mL of CSF using amplicon next-generation sequencing approaches (NGS) methodology (SafeSeqS).^16^ Similar results have been observed in terms of detectable mutations in ctDNA derived from CSF in patients with pHGG and DIPG by using nested polymerase chain reaction (PCR), droplet digital PCR (ddPCR), and panel sequencing.^21–24^ These studies have also demonstrated that CSF-derived ctDNA levels increase during disease progression.^22,23^ In adult glioma, use of the MSK-IMPACT capture-based NGS assay identified ctDNA in CSF from around half of patients, with ctDNA levels correlating with disease burden and poor outcome.^25^

Less is known about the utility of plasma-derived DNA from brain tumors toward mutation detection and treatment response monitoring, and in particular in pHGG, DIPG, and other DMG. Pan and colleagues showed the detection of ctDNA by custom capture panel sequencing, derived from 3 mL of plasma in 3/8 pediatric patients with brainstem tumors; of those three, two had undetectable mutations in the plasma ctDNA compared to the ctDNA derived from the CSF.^24^ Conversely, a study from Panditharatna and colleagues showed detectable levels of ctDNA derived from 1 mL of plasma in 16/20 patients with DMG at diagnosis using ddPCR.^23^ Notably, a recent publication described the use of cell-free methylated DNA immunoprecipitation and high-throughput sequencing (cfMeDIP-seq) on ctDNA derived from plasma in a range of adult glioma specimens.^26^ Given the importance of methylation-based profiling for subtyping pediatric diffuse glioma, this would be an important technique to assess in the childhood context.

The implementation of ctDNA from plasma and CSF in routine clinical practice represents an important goal for the field. The inclusion criteria for an increasing number of clinical trials require molecular characterization to confirm biomarker positivity. For example, *H3F3A*_K27M and *BRAF*_V600E need to be confirmed in tumors for patients to be eligible for current clinical trials of ONC201 (NCT03416530) or dabrafenib in combination with trametinib (NCT02684058), respectively. This is of particular importance for patients such as those with DIPG, where tissue biopsy remains an invasive procedure, not without complications.^3,4^ In addition, the analysis of ctDNA can provide a unique opportunity to assess therapeutic response to a targeted agent, as well as to track tumor evolution in response to therapy, and to identify potential resistance mechanisms that may inform novel treatment options at relapse.

To this end, we sought to explore whether molecular alterations could be identified in liquid biopsy samples from pHGG, DIPG, and other DMG patients. ddPCR assays were validated and applied to quantify ctDNA levels derived from plasma, serum, and CSF. We also explored whether circulating DNA concentrations correlated with tumor burden and multimodal radiological indicators of response and tumor progression using samples collected within a clinical trial in nonbrainstem pHGG/DMG.

## Methods

### Cases

All patient samples were collected after signed consent to either the HERBY or BIOMEDE translational research programs, or local Institutional Research Board, under full Research Ethics Committee approval at each participating center. A total of 44 samples from different source of liquid biopsy sample sources (plasma *n* = 27, serum *n* = 6, CSF *n* = 10, and Cyst fluid *n* = 1), were collected from local studies (Royal Marsden Hospital *n* = 26) and collaborators (Ospedale Pediatrico Bambino Gesù *n* = 8 and BIOMEDE *n* = 10). Samples corresponding to 33 patients harbored mutations identified in the tissue tumor sample by next-generation sequencing (whole-exome sequencing and capture panel sequencing assays^27–29^). In addition, 127 plasma aliquots from different time-points, were available from 41 HERBY patients, harboring driving mutations identified by whole-exome-sequencing of pre-treatment tissue tumor samples.^29^

### Liquid Biopsy Samples

Where possible, up to 10 mL of peripheral blood was collected into Cell-Free DNA Collection Tubes (Streck, La Vista). Samples were centrifuged twice for 10 min, first at 1600 g and at up to 16,000 g to remove cellular contents and/or debris. Samples were stored at −80°C until cfDNA extraction. Local protocols to isolate plasma and CSF were used for the remaining liquid biopsies cases, collected from different sources. cfDNA isolation from plasma and CSF supernatant was performed using the QIAamp circulating nucleic acid kit (Qiagen, 55114) following quantification using the Qubit fluorometer (ThermoFisher Scientific, dsDNA HS Assay kit, Q32854) and fragment analysis by 2200 and 4200 TapeStation (Agilent, Genomic DNA ScreenTape 5067–5366).

### Droplet Digital PCR

Custom TaqMan-based quantitative PCR genotyping assays (Applied Biosystems, Thermo Scientific and IDT, Integrated DNA Technologies) were designed to specifically detect genetic abnormalities (mutations) (Supplementary Table S2). Commercially available assays were used to identify *MYCN* amplification (*MYCN* Hs00201049_cn, control region 4403316 or 4403326, Applied Biosystems, Thermo Scientific) and *H3F3A*_K27M (*H3F3A*_K27M dHsaCP2500510; *H3F3A*_WT dHsaCP2500511, Bio-Rad) as well as *H3F3A*_G34R (*H3F3A*_G34R dHsaIS2502308; *H3F3A*_WT dHsaIS2502309, Bio-Rad). The assay limit of detection (LoD) was assessed by performing serial dilutions of the mutant DNA in a constant concentration of wild-type DNA (1:10, 1:100, 1:1000 and 1:10,000) and run in duplicate using 5 ng of DNA. The LoD was calculated as the fractional abundance of the neat mutant sample divided by the lowest dilution with detectable mutant copies (at least two mutant droplets).^30,31^ For each assay, three controls were run in duplicates including: one non-template control, one wild-type control (fragmented Promega DNA at 1 ng/µL), and one positive control harboring the alteration of interest.

The Bio-Rad QX200 ddPCR system was used, which allows the detection of rare DNA target copies with high sensitivity. DNA was randomly encapsulated into approximately 15,000 oil nanoliter-sized droplets, using the Automated Droplet Generator (BioRad, QX200 AutoDG), containing ddPCR Supermix for probes (no dUTP) (BioRad, 1863024), genotyping assay (specific per alteration), water, and the DNA of interest. The PCR reaction was performed in a thermocycler plates were then placed on the droplet reader where the droplets are streamed individually through a detector and signals from mutant positive (FAM), wild-type (VIC/HEX), double-positive (FAM and VIC/HEX), and negative droplets (empty) are counted to provide absolute quantification of DNA in digital form. The mutant allele concentration (C_MUT_) and wild-type allele concentration (C_WT_) were calculated with Quantasoft Analysis Pro (BioRad), the mutant allele fraction (AF_dPCR_) and the concentration of cfDNA in the CSF or plasma (CcfDNA ng/mL) were calculated with the following calculations as previously described in ^17,18,22^:


$$\begin{array}{l} AF_{dPCR}=\;C_{MUT}/\left(C_{MUT}+C_{WT}\right) \end{array}$$

$$\begin{array}{l} C_{MUT\_ORI}=V_{PCR}\times C_{MUT}\times V_{ELU}/V_{DNA-PCR}\times V_{SAMPLE} \end{array}$$

$$\begin{array}{l} C_{WT\_ORI}=V_{PCR}\times C_{WT}\times V_{ELU}/V_{DNA-PCR}\times V_{SAMPLE} \end{array}$$

*CMUT_ori is mutant allele concentration in original CSF or plasma (copies/mL)*

*CWT_ori is wild-type allele concentration in original CSF or plasma (copies/mL)*

*VPCR is the volume of final PCR mix (μL)*

*VSAMPLE is the volume of CSF or plasma used to extract cfDNA (mL)*

*VELU is the volume of cfDNA elution generated from DNA extraction (μL)*

*VDNA-PCR is the volume of cfDNA used in the final PCR mix*


$$\begin{array}{l} CcfDNA\approx 0.003\times \left(C_{MUT}+C_{WT}\right) \end{array}$$

*the mass of 1 haploid human genome is 0.003 ng*

### Fusion Panel

A custom fusion panel consisting of 24 genes associated with fusions in pediatric brain tumors (*ALK, BCOR, BEND2, BRAF, c11orf95, C19MC, CIC, ETV6, FGFR1-3, FOXR2, QKI, KIAA1549, MET, MN1, MYB, MYBL1, NTRK1-3, RAF1, RELA, TPM3, and YAP1*) was designed with a library of probes to ensure adequate coverage of the specified regions (Roche Sequencing Solutions).^29^ 30 ng of cfDNA was used for library preparation using KAPA HyperPlus Kit (Kapa Biosystems) and SeqCap EZ adaptors (Roche) without performing the fragmentation step. DNA was end-repaired, A-tailed, and indexed adaptors ligated, amplified, multiplexed, and hybridized using 1 μg of the total pre-capture library DNA. After hybridization, capture libraries were amplified and sequencing was performed on a MiSeq (Illumina). Quality control (QC), variant annotation, deduplication, and metrics were generated for each sample. The raw list of candidates provided by Manta (https://github.com/Illumina/manta) were filtered for more than 2 reads covering both genes, common false-positive base pairs (bp) positions/fusions outside of the capture set at both ends, common breakpoint/false-positives within 10 bp, common false positive gene pairs, fusions within the same gene and homologous sequences greater than 10 bp.

### Radiological Evaluation

Analysis of tumor burden from the HERBY cohort was carried out at different time-points based on imaging and clinical data. Following image review by up to three expert pediatric neuroradiologists on the HERBY Central Radiology Committee using the Response Assessment in Neuro-Oncology (RANO) criteria,^32^ an independent pediatric oncologist reviewed supportive clinical data and corticosteroid dosage and provided the final status for that time point.^[29,30]^

### Statistical Analysis

Statistical analysis was carried out using GraphPad Prism 8, using one-way ANOVA with multiple testing correction. An adjusted *P*-value of less than .05 was considered significant.

## Results

### ddPCR Assay Validation for the Detection of ctDNA from Liquid Biopsies

**Figure 1.**
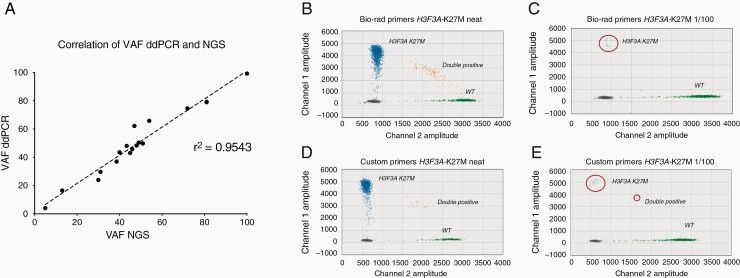
*ddPCR assay validation.* (A) Correlation of variant allele frequencies (VAFs) by NGS (x-axis) and ddPCR (y-axis) for ddPCR assay validation. 13 assays were tested in samples positive for the mutations analyzed (n = 18). A linear regression is fitted, with a Pearson correlation coefficient calculated and labeled, r^2^ = 0.9543. (B) Droplet digital PCR 2D amplitude plot of *H3F3A*_K27M tested in a positive control DIPG sample using the Bio-Rad assay on undiluted (neat) *H3F3A*_K27M DNA (1760/2226 VAF of 79.3%). (C) Bio-Rad assay on 1/100 dilution of *H3F3A*_K27M DNA with wild-type DNA (10/1564 droplets, VAF of 6.4%). (D) Custom assay on neat *H3F3A*_K27M DNA (1586/2014 VAF of 79.1%). (E) Custom assay on 1/100 dilution of *H3F3A*_K27M DNA with wild-type DNA (17/1613 droplets, VAF of 7.7%). *H3F3A*_K27M droplets are shown in blue, H3.3 wild-type droplets are shown in green, double positive droplets are shown in orange and empty droplets with no DNA are shown in grey.

Liquid biopsies from multiple biological sources (plasma, serum, cerebrospinal fluid [CSF], and cystic fluid) were collected from 32 pHGG and DIPG patients with known molecular alterations from the sequencing of their tumor tissue. These patients harboured somatic mutations in *H3F3A* (K27M and G34R), *BRAF* (V600E), *ACVR1* (G328V), *IDH1* (R132H and R132S), *TP53* (C238Y and R282W), and *PIK3CA* (E542K and H1047R), and one had *MYCN* amplification. The first goal was to develop a robust detection method for these genetic alterations. To do this, customized and commercially available assays for ddPCR were validated for the identification of patient-specific molecular alterations. Each genotyping assay was tested by using a positive sample harboring the specific alteration of interest, and the variant allele frequency (VAF) was compared between ddPCR and NGS, with an observed correlation of *r*^2^ = 0.9543 (Figure 1A).

To assess the limit of detection (LoD) of point mutation detection assays, mutant DNA samples were serially diluted 10-fold in wild-type genomic DNA (1/10, 1/100, 1/1000, and 1/10,000). Genomic DNA from tissue was fragmented and a total DNA input of 5 ng was utilized to simulate the anticipated low amount of ctDNA. LoD was calculated as the VAF of the neat sample divided by the lowest dilution with detectable signal for mutant, with at least two droplets containing mutant DNA. Two different *H3F3A*_K27M assays were assessed, one commercially available from Bio-Rad and one reported by Stallard and colleagues.^22^ Both assays performed well, obtaining a good droplet separation between FAM and VIC/HEX labels, with a similar LoD (Bio-Rad = 0.793% and custom = 0.791%) (Figure 1B–E). In addition, no mutant droplets were observed in any of the wild-type template control DNA included per assay in each run. By using 5 ng of DNA, LoD ranged from 0.041% (*PIK3CA*_E542K) to 0.993% (*TP53*_C238Y), with a median of 0.203% (Supplementary Table S1) and (Supplementary Figure S1).

The *MYCN* amplification assay contained two probes, one within the *MYCN* gene and one in a control region at chromosome 5p15.33. The amplification assay was tested by comparing the ratio of copies/µL of *MYCN* to the control gene (Supplementary Figure S2A). Two ctDNA-plasma positive samples from *MYCN*-neuroblastoma patients were used for the assay validation, with tissue samples for each patient used as a positive control and run in duplicate. *MYCN* amplification was detected in the DNA derived from the tissue (Supplementary Figure S2B,D) and the ctDNA isolated from plasma (Supplementary Figure S2C,E). The ctDNA samples taken at diagnosis from the two patients showed a fold-amplification of 32 and 110.

### Genetic Alterations can be Detected in ctDNA from Liquid Biopsies of pHGG and DIPG Patients

**Figure 2.**
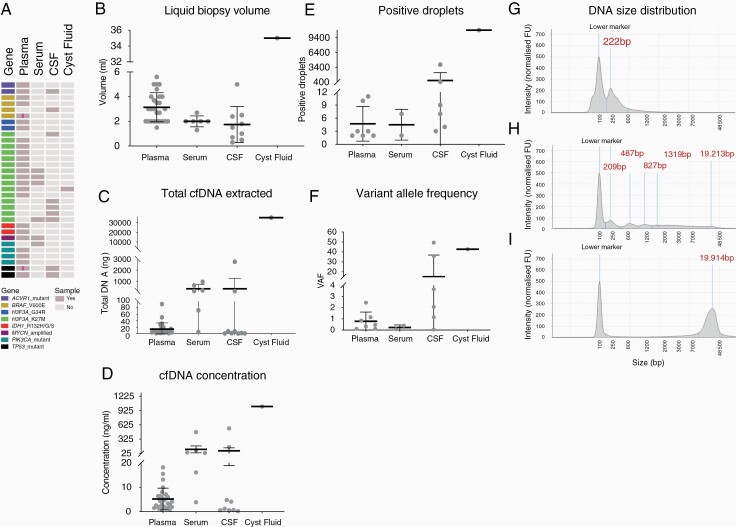
Detection of genetic alterations in ctDNA from pHGG and DIPG patients. (A) A cohort of pHGG and DIPG samples used for liquid biopsy feasibility study, with each row representing a patient and each column a sample. Cells are colored by molecular alteration assessed and sample availability according to the key provided. A pink line indicates multiple samples for that case. (B) Dot plot of the volume of liquid biopsy used for cfDNA extraction, separated by the biological source material. Each sample is represented by a dot, the middle line represents the median, and the upper and bottom line the standard deviation. (C) Dot plot of total cfDNA extracted from liquid biopsy samples. Each sample is represented by a dot, the middle line represents the median, and the upper and bottom line the standard deviation. (D) Dot plot of cfDNA concentrations of liquid biopsy samples, separated by the biological source material. Each sample is represented by a dot, the middle line represents the median, and the upper and bottom line the standard deviation. (E) Dot plot of positive (>2) ddPCR droplets from liquid biopsy samples, separated by the biological source material. (F) Dot plot of variant allele frequency (VAF) for ctDNA samples, separated by biological source material. Each sample is represented by a dot and the middle line represents the mean. (G) Electropherogram of cfDNA size distribution was obtained by using the TapeStation for a representative serum sample (B118) showing a smear indicating a high degree of genomic DNA fragmentation. (H) Electropherogram of cfDNA size distribution obtained by using the TapeStation for a representative CSF sample (045-T) with a prominent ctDNA peak with an average size of 222 bp. (I) Electropherogram of cfDNA size distribution obtained by using the TapeStation for a representative CSF sample (C15-654) with intact genomic DNA contamination with an average size of 19,914 kb. The *y*-axis shows the signal intensity (FU) and the *x*-axis shows the DNA fragment size is represented in base pairs (bp).

To test the feasibility of ctDNA detection in pHGG and DIPG, the validated ddPCR methodology was applied in a cohort of 43 liquid biopsy samples from the 32 patients, which included plasma (*n* = 27), serum (*n* = 6), CSF (*n* = 9) and cyst fluid (*n* = 1) (Figure 2A). The average volume of fluid obtained was 3.14 mL (SD = 1. 2) for plasma, 2 mL (SD = 0.4) for serum, and 1.74 mL (SD = 1.5) for CSF (Figure 2B). From patient 131-T, a large volume (350 mL) of cystic fluid was collected at time of resection and 35 mL were used for cfDNA extraction. The mean cfDNA concentration was 5.2 ng/mL (SD = 4.4) from plasma samples, 110.8 ng/mL (SD = 179.9) from serum and 80.33 ng/mL (SD = 184.2) from CSF (Figure 2C). 1012 ng/mL were obtained from the cyst fluid sample.

Molecular alterations were found in a total of 16 ctDNA samples, including those derived from plasma (7/27, 26%), CSF (6/9, 67%), serum (2/6, 33%) and the only cystic fluid specimen available. Variants included *H3F3A*_G34R (*n* = 2), *H3F3A*_K27M (*n* = 7), *IDH1*_R132H (*n* = 1), *PIK3CA*_H1047R (*n* = 1), *PIK3CA*_E542K (*n* = 1), *ACVR1*-G328V (*n* = 1), *TP5*3_C238Y (*n* = 1), and *TP53*_R282W (*n* = 2) (Table 1). Although not formally significant due to small numbers and high degree of variability, the average of positive droplets was higher in ctDNA derived from CSF (median = 735.7, SD = 1582), than from plasma (median = 4.7, SD = 3.9) and serum (median = 4.5, SD = 3.53) (*P* = .5879 and *P* = .8167, respectively, one-way ANOVA, Tukey’s multiple comparisons test) (Figure 2D). Similarly, the average VAF was higher in ctDNA derived from CSF (median = 15.33%, SD = 21.54%) than from plasma (median = 0.78%, SD = 0.31%) and serum (median = 0.22%, SD = 0.16%) (*P* = .2867 and *P* = .5633, respectively, one-way ANOVA, Tukey’s multiple comparisons test) (Figure 2E). The highest number of positive droplets (10,944, VAF = 42.68%) was found in the cystic fluid. More than two-thirds (70%) of samples from all biosources in which there were no detectable alterations were from DIPG or DMG patients. Paired CSF/cyst fluid and plasma/serum were available for five patients—of these, two alterations were detected in both liquid biopsy sources and for the remaining three cases variants were only identified in the CSF (all of whom were also DIPG or DMG). For patient 045-T, who presented with a hemispheric HGG with hypermutator phenotype (210 mutations per Mb), *TP53*_R282W was identified in ctDNA derived from CSF (VAF = 49.34%) and the plasma (VAF = 0.12%). In addition, patient-131-T, with a right thalamic glioma, *H3F3FA_*K27M was identified in the cystic fluid (VAF = 42.68%) and the plasma (VAF = 0.85%). Although the formal threshold for a positive sample was set as at least two positive droplets, a single positive droplet was found in seven cases, including five cfDNA derived from plasma (*H3F3FA_*K27M *n* = 4, and *ACVR1*_G328V *n* = 1) and two CSF (*H3F3FA_*K27M *n* = 2).

**Table 1.** Summary of Liquid Biopsy Samples With Detectable ctDNA From pHGG/DIPG Patients

Patient ID	ddpcr Assay ID	SAMPLE type	Volume for ctDNA	VAF	Mutant Droplets	Wild-type Droplets	cfDNA Mutant ng/mL	cfDNA Wild-Type ng/mL
013-T	*H3F3A* -K27M	Plasma	2.7	2.451	10	398	0.028	1.131
045-T	*TP53*-R282W	CSF	5	49.343	3945	4050	271.913	279.585
045-T	*TP53*-R282W	Plasma	5	0.118	3	2540	0.021	18.313
054-T	*H3F3A*-G34R	Plasma	5	0.468	2	425	0.007	1.561
106-T	*IDH1*-R132H	Plasma	3.5	0.335	11	3272	0.029	8.844
120-T	*H3F3A*-G34R	Plasma	3	1.143	2	173	0.005	0.422
131-T	*H3F3A* -K27M	Cyst	38.5	42.68	10944	14698	367.594	541.671
131-T	*H3F3A* -K27M	Plasma	2	0.85	3	350	0.009	1.048
15–3381	*H3F3A* -K27M	CSF	1	3.646	7	185	0.044	1.177
16120B	*PIK3CA*-E542K	Serum	2	0.072	7	9674	0.291	472.795
ICR-B134	*H3F3A* -K27M	Serum	1.3	0.39	2	511	0.015	3.934
ICR-B276	*PIK3CA*-H1047R	Plasma	2	0.116	2	1727	0.006	5.098
ICR-CXJ-026	*TP53*-C238Y	CSF	2.5	2.073	4	189	0.013	0.633
ICR-CXJ-028	*ACVR1* G328V	CSF	1.8	35.737	446	802	1.472	2.662
I-16–3200	*H3F3A* -K27M	CSF	0.7	0.051	3	5916	0.071	159.274
I-16–855	*H3F3A* -K27M	CSF	1.5	1.149	9	774	0.054	4.703

Shown are the mutations assessed by ddPCR, the biological source material, volume used for the ddPCR assay, variant allele frequency (VAF), number of mutant and wild-type droplets as well as concentration of mutant and wild-type cfDNA.

By assessing the DNA integrity with a TapeStation electrophotometric analyzer, ctDNA was found in 8/13 samples with detectable cfDNA (Figure 2F). Of note, 4/6 cfDNA extracted from serum presented a smear of fragmented DNA including genomic DNA (Figure 2G), whilst one CSF sample (I-16–3200, *H3F3A*-K27M positive), had a pronounced gDNA contamination and had the lowest VAF of CSF-ctDNA samples (0.05%) (Figure 2H).

Finally, a custom pediatric brain tumor fusion panel^29,33^ was used to detect a known *ETV6:NTRK3* fusion in the CSF from a single infant glioma patient (OPBG_INF_035). 30 ng of cfDNA extracted from 4.5 mL of CSF was run on the capture panel, with 23 reads supporting the fusion detected (Supplementary Figure S3).

### Exploring the Use of Liquid Biopsy in the HERBY Clinical Trial Cohort

**Figure 3.**
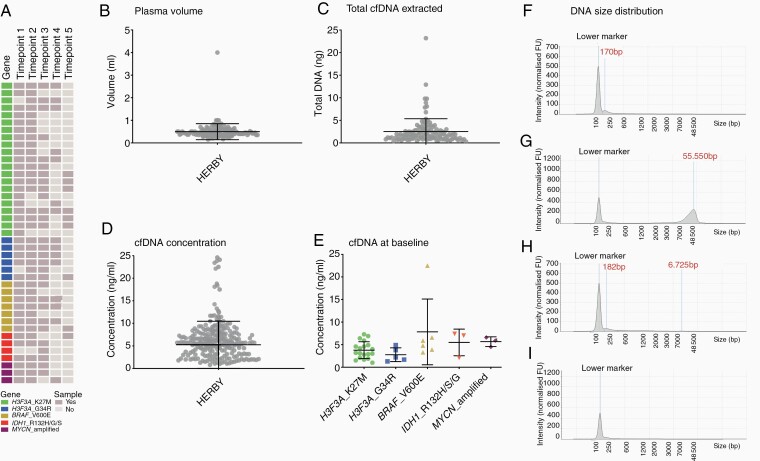
Quantitation of plasma samples collected as part of the HERBY clinical trial. (A) A cohort of nonbrainstem pHGG plasma samples from the HERBY clinical trial, with each row representing a patient and each column a sample. Cells are colored by molecular alteration assessed and sample availability according to the key provided. Time-points are as follows: 1 = baseline, 2 = week 3, 3 = week 7, 4 = month 6, 5 = end of treatment. (B) Dot plot of volume of plasma used for cfDNA extraction. Each sample is represented by a dot, the middle line represents the median, and the upper and bottom line the standard deviation. (C) Dot plot of total cfDNA extracted from HERBY plasma samples. Each sample is represented by a dot, the middle line represents the median, and the upper and bottom line the standard deviation. (D) Dot plot of cfDNA concentration per mL from HERBY plasma samples. Each sample is represented by a dot, the middle line represents the median, and the upper and bottom line the standard deviation. (E) Dot plot of cfDNA concentration at baseline, separated by molecular subgroup. Each sample is represented by a dot, the middle line represents the median, and the upper and bottom line the standard deviation. (F) Electropherogram of cfDNA size distribution was obtained by using the TapeStation for a representative plasma sample with detectable levels of cfDNA (~170 bp). (G) Electropherogram of cfDNA size distribution was obtained by using the TapeStation for a representative plasma sample with a high degree of genomic DNA contamination (>55 kb). (H) Electropherogram of cfDNA size distribution obtained by using the TapeStation for a representative plasma sample with detectable cfDNA (182 bp) and genomic DNA (~6.7 kb) peaks. (I) Electropherogram of cfDNA size distribution obtained by using the TapeStation for a representative plasma sample with detectable DNA. The y-axis shows the signal intensity (FU) and the x-axis shows the DNA fragment size is represented in base pairs (bp).

To assess the utility of liquid biopsies for molecular diagnostics and to monitor disease progression, we studied genetic alterations in cfDNA derived from plasma from longitudinal samples from the well-annotated HERBY trial in non-brainstem pHGG (NCT01390948).^29,34–37^ Blood samples were taken at up to five different time-points during the course of treatment, with plasma isolated locally and sent to our laboratory. cfDNA was extracted from 127 plasma samples from 41 patients, selected for tumors harboring alterations in *H3F3A, IDH1*, *BRAF,* or *MYCN* (Figure 3A). The mean volume of plasma from which cfDNA was extracted was 0.49 mL (SD = 0.35, excluding one sample from which 4 mL of plasma were used for extraction) (Figure 3B).

The mean yield of total DNA extracted from plasma was 2.52 ng (SD = 2.83, excluding the four cases with high levels of genomic DNA) (Figure 3C). The mean of total DNA yield extracted per mL of plasma was 5.25 ng (SD = 5.21, excluding the four cases with high levels of genomic DNA) (Figure 3D). The DNA samples were run undiluted and the mean of DNA ddPCR input was 1.76 ng (SD = 2.04). Disappointingly, none of the HERBY cfDNA samples tested for the known genetic alterations were positive (>two mutant droplets for point mutations and >4-fold for *MYCN* amplification). However, there were four cases where one positive droplet was found (*BRAF*_V600E, *n* = 2; *H3F3A*_K27M, *H3F3A*_G34R, *n* = 1 each). All four patients received bevacizumab and had stable disease as their best radiological response. cfDNA concentration was compared between molecular subgroups. Although there was no significant difference between subgroups at baseline (*P* = .1026, one-way ANOVA), there was a trend of higher concentration of cfDNA in *BRAF*_V600E positive patients compared to *H3F3A*_K27M and *H3F3A*_G34R (*P* = .0547 and *P* = .0661, respectively, one-way ANOVA, Dunnett’s multiple comparisons test) (Figure 3E). DNA integrity was measured by using TapeStation, showing four different types of DNA size distribution: 33 samples presented a detectable cfDNA peak (~170 bp) (Figure 3F), five samples contained a high amount of genomic DNA contamination (>55 kb) (Figure 3G), 12 samples showed detectable cfDNA and genomic DNA peaks (Figure 3H), whilst in the remaining 75 samples no DNA was detectable (Figure 3I).

**Figure 4.**
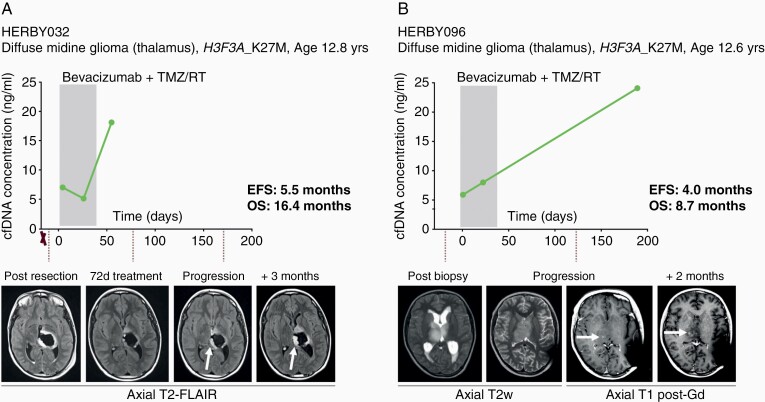
Correlation of plasma cfDNA concentration and poor response in DMG-K27M. (A) HERBY032, diffuse midline glioma, WHO grade IV *H3F3A*_K27M mutant, event-free survival (EFS) of 5.5 months, overall survival (OS) of 16.4 months. cfDNA concentrations (y axis) plotted against time from randomization (days). Resections are marked with an X. Below, axial T2-weighted Fluid and Attenuation Inversion Recovery (FLAIR) MRI scans at different time-points of the patient’s disease, with white arrows highlighting an enlarging hyperintense abnormality at the cavity margins. The shaded box represents the initial 6-week treatment of RT/TMZ with bevacizumab. Subsequent to this, there were repeated cycles of TMZ every 28 days, and bevacizumab every 2 weeks, until the end-point. (B) HERBY096, diffuse midline glioma, WHO grade IV, *H3F3A*_K27M mutant, EFS of 4 months, OS of 8.7 months. cfDNA concentrations (y axis) plotted against time from randomization (days). Resections are marked with an X. Below, axial T2-weighted or T1 post-gadolinium MRI scans at different time-points of the patient’s disease, with white arrows highlighting a new focus of enhancement. The shaded box represents the initial 6-week treatment of RT/TMZ with bevacizumab. Subsequent to this, there were repeated cycles of TMZ every 28 days, and bevacizumab every 2 weeks, until the end-point.

Finally, although we were not able to reliably detect ctDNA in the HERBY plasma samples, we explored the correlation of cfDNA concentrations to disease burden and tumor progression. When assessing the changes in cfDNA concentrations over the course of the individual patient’s disease (Supplementary Figure S4), anecdotal variations across longitudinal time-points were observed in four patients. Two DMGs, both *H3F3A*_K27M mutated, exhibited increased cfDNA concentrations at later timepoints, corresponding with a relatively short time to progression in these cases. The first (HERBY032) was a 12.8 year-old boy who underwent a near-total resection prior to treatment with bevacizumab and chemoradiotherapy. He displayed local recurrence at 5.5 months, though there was a marked increase in cfDNA concentration 3 months earlier. He died at 16.4 months postrandomization (Figure 4A). The second (HERBY096) was a 12.6 year-old boy, also on the bevacizumab arm, but who was eligible for biopsy only, and had local progression at 4.0 months. There was a substantial cfDNA increase in the subsequent plasma sample two months later, and he died of disease at 8.7 months (Figure 4B).

**Figure 5.**
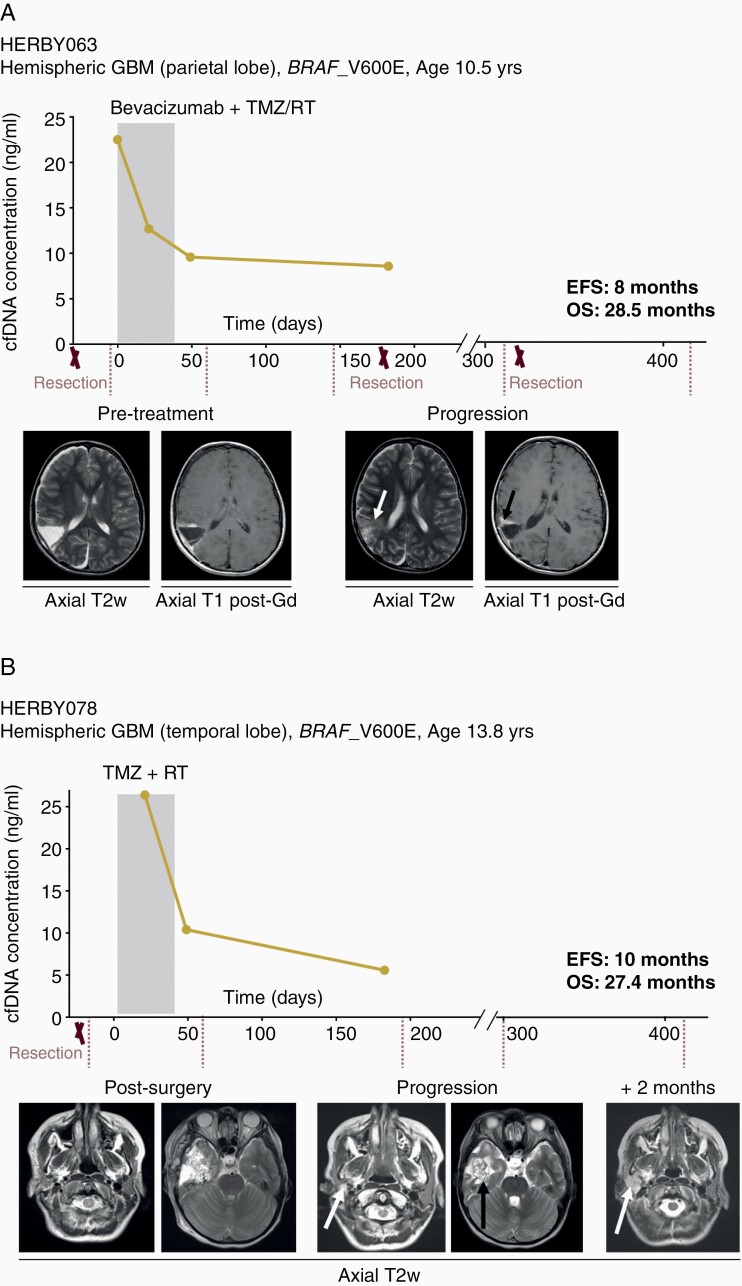
Correlation of plasma cfDNA concentration and better outcome in hemispheric BRAF_V600E mutant GBM. (A) HERBY063, hemispheric glioblastoma, WHO grade IV, *BRAF*_V600E mutant, event-free survival (EFS) of 8 months, overall survival (OS) of 28.5 months. cfDNA concentrations (y axis) plotted against time from randomization (days). Resections are marked with an X. Below, axial T2-weighted or T1 post-gadolinium MRI scans at different time-points of the patient disease, with white arrow highlighting an increased T2 abnormality, and the black arrow showing the progressive enhancing tumor. The shaded box represents the initial 6-week treatment of RT/TMZ with bevacizumab. Subsequent to this, there were repeated cycles of TMZ every 28 days, and bevacizumab every 2 weeks, until the end-point. (B) HERBY078, hemispheric glioblastoma, WHO grade IV, *BRAF*_V600E mutant, EFS of 10 months, OS of 27.4 months. cfDNA concentrations (y axis) plotted against time from randomization (days). Resections are marked with an X. Below, axial T2-weighted MRI scans at different time-points of the patient disease, with white arrows highlighting a new parotid lesion, and the black arrow indicating the primary site recurrence. The shaded box represents the initial 6-week treatment of RT/TMZ. Subsequent to this, there were repeated cycles of TMZ every 28 days until the end-point.

Conversely, two hemispheric glioblastomas with *BRAF*_V600E mutations showed a reduction in cfDNA concentration from baseline and early time-points, corresponding to longer progression-free survival. HERBY063 was a 10.5-year-old boy who underwent three resections and survived for 28.5 months postrandomization to bevacizumab plus chemoradiotherapy. There was a marked decrease in cfDNA concentration at the earliest timepoints in the first two months, and prior to later local recurrence with slow growth at 8 months (Figure 5A). Finally, HERBY078 was a 13.8-year-old girl treated with temozolomide and radiotherapy alone, and also displayed a substantial initial decrease in cfDNA. She progressed at 10 months locally and below the skull base, with evidence of parotid gland metastatic spread. Nonetheless, she survived on treatment for 27.4 months before succumbing to her disease (Figure 5B).

## Discussion

This study describes the validation of a number of ddPCR assays for the detection of point mutations in cfDNA. These include key genes commonly altered in pHGG, DIPG, and other DMG, including *H3F3A, IDH1, PIK3CA, BRAF, ACVR1*, and *TP53,* as well as amplification of *MYCN*. By applying this methodology to cfDNA, tumor mutations were detectable in CSF, cystic fluid, plasma, and serum derived from pHGG and DIPG patients. In accordance with other studies, it was found that ctDNA was present at a higher percentage and with greater VAFs in cfDNA derived from CSF compared to plasma and/or serum specimens (~67% compared to 26% and 33% samples, respectively); these data support the use of CSF over plasma as source of tumor DNA for molecular profiling.^17,19–21,24,25,38^ The detection range of ctDNA in CSF has been fairly consistent among studies, ranging from 66 to 84%,^21,23,24^ whilst there is little concordance for plasma samples (16–80%).^20,23–25,39^

Circulating tumor DNA represents a small fraction of total cfDNA, and the low yields seen in pHGG and DIPG patients represent a major challenge for the detection of this potentially useful biomarker. It is thought that the low permeability of the brain–blood barrier might prevent ctDNA from spreading into the bloodstream. This is supported by the fact that higher ctDNA levels derived from plasma are observed in patients with diffuse midline glioma after radiation (72–100 hours), suggesting that radiotherapy might disrupt the BBB allowing ctDNA to be released into the bloodstream.^22,23^ Another possible reason for lower levels of ctDNA isolated from plasma and or serum is the presence of background genomic DNA from non-malignant cells. In particular, it was observed that no ctDNA was detected in samples presenting highly fragmented cellular DNA, presumably derived from cells undergoing necrosis. Samples presenting higher levels of genomic DNA in our cohorts were mostly derived from external institutions where blood samples were not taken using collection tubes containing a preservative stabilizer of nucleated blood cells such as Streck or PAXgene blood ccfDNA tubes. The use of these tubes is highly recommended to prevent cell lysis, and when this is not possible samples taken in EDTA tubes should be processed within 2 h of blood withdrawn.

Longitudinal plasma samples from HERBY, the largest randomized clinical trial in non-brainstem pHGG, represented a unique cohort to test the utility of such approaches for disease monitoring.^29,34–37^ Unfortunately, at the time of study initiation in 2011, the protocol allowed for only small fluid volumes to be taken, as liquid biopsy approaches had not yet been considered, and it is unfortunate that no ctDNA could be detected from such limited amounts. This is an important consideration for future trials, with at least 4 mL of plasma required for liquid biopsy tests used in clinical practice such as Guardant360.^40^ Despite this, cfDNA concentrations themselves, when detectable above baseline, correlated with early disease progression and poor outcome in two patients with K27M mutated DMG, and a better outcome for two patients with *BRAF*_V600E mutated hemispheric GBM patients.

As cellular DNA contamination can affect the sensitivity of ctDNA detection, some studies have applied in-silico and in vitro size selection to a achieve higher sensitivity evaluation of ctDNA.^41^ However, this needs to be further verified as size selection after cfDNA extraction might contribute to potential loss of ctDNA material. Another strategy that Panditharatna and colleagues used in their study, which detected ctDNA in 80% of diffuse midline gliomas at diagnosis/upfront therapy, was a pre-amplification step of 9 cycles.^23^ This could explain their high detection rate and should be further validated to assess the potential false positive rate introduced by pre-amplification. Newer strategies combining the use of unique molecular identifiers (UMIs), to facilitate the identification of single DNA molecules from PCR duplicates, with deep sequencing, are promising strategies to detect ctDNA.^42,43^ In addition, this strategy sequences a list of genes that can be customized allowing the detection of multiple genes, which can be valuable to track emergence of resistance alterations. In this context, Cell3 Target (Nonacus, oncology) offers calling of mutations down to 0.1% of VAF from as little as 10 ng ctDNA input by incorporating UMIs into targeted NGS customised gene panel.

In summary, we could identify tumor-specific DNA alterations more readily in CSF than plasma, demonstrating the feasibility of tracking tumor response, but also highlighting the importance of sufficient plasma volumes and additional techniques that could enhance yield in these samples. This is particularly critical to avoid the risks associated with repeated sampling of CSF for serial monitoring over time in children with this disease.

## Supplementary Material

vdab013_suppl_Supplementary_Materials

## Notes

**Authorship Statement** E.I., M.H., and C.J. conceived the study. E.I. and P.P. carried the limit of detection assays and analyzed data. E.I., S.T., and M.C. carried out molecular profiling. E.I., D.C., and A.M. carried out molecular analysis. L.V.M., F.C., D.H., B.L., Z.P., S.B., N.E.-W., J.G., G.V., and M.V. provided samples and clinical annotation. D.R., P.S.M., and T.J. carried out a radiological evaluation from the HERBY clinical trial. E.I. and C.J. wrote the manuscript.
