# Supplementary material for: Droplet digital PCR-based detection of circulating tumor DNA from pediatric high grade and diffuse midline glioma patients
Source: Neurooncol Adv. 2021 Jan 27;3(1):vdab013. doi: 10.1093/noajnl/vdab013 (PMC8218704; doi:10.1093/noajnl/vdab013)
Supplement: vdab013_suppl_Supplementary_Materials [file vdab013_suppl_supplementary_materials.docx]

**SUPPLEMENTARY FIGURE LEGENDS**

**
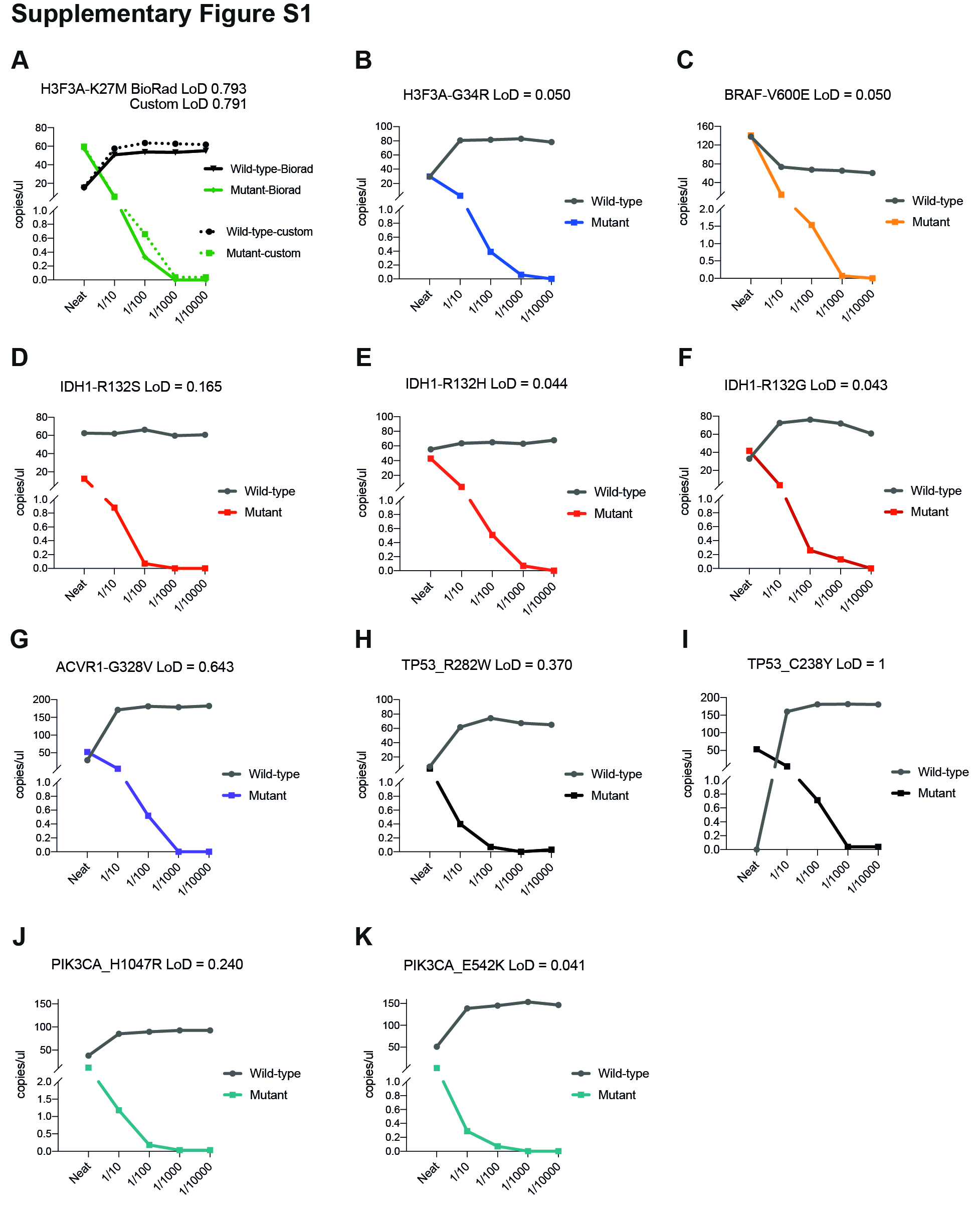
**

**Supplementary Figure S1** - *ddPCR assay limit of detection plots.* Linear dilutions of mutant DNA in a constant background of wild-type DNA are shown against number of copies/µl for wild-type and mutant alleles. A total of 5 ng of total DNA was load in each PCR well. Samples were run in duplicates and merged data is shown. (A) *H3F3A*_K27M (green), (B) *H3F3A*_G34R (blue), (C) *BRAF*_V600E (gold), (D) *IDH1*_R132S (red), (E) *IDH1*_R132H (red), (F) *IDH1*_R132G (red), (G) *ACVR1*_G328V (purple), (H) *TP53*_R282W (grey), (I) *TP53*_C238Y (grey), (J) *PIK3CA*_H1074R (teal), (K) *PIK3CA*_E542K (teal).

**
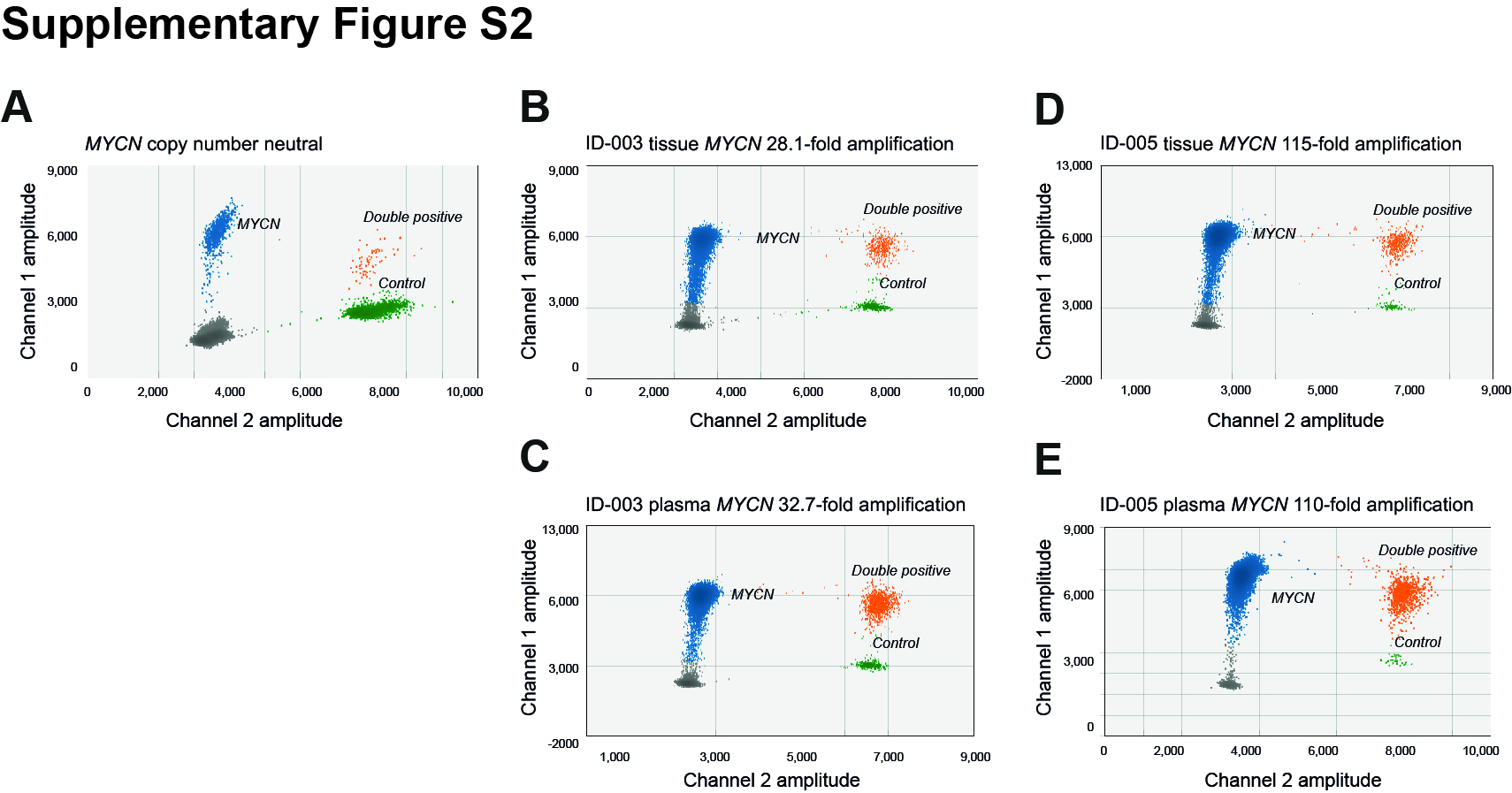
**

**Supplementary Figure S2** - *ddPCR assay validation.*

(A) Droplet digital PCR 2D amplitude plot of *MYCN* tested in a patient with copy neutral *MYCN*, and (B,D) tissue and (C,E) plasma from two positive control neuroblastoma patients with known *MYCN* amplification. *MYCN* droplets are shown in blue, droplets from a control region at chromosome 5p15.33 are shown in green, double positive droplets are shown in orange and empty droplets with no DNA are shown in grey.

**
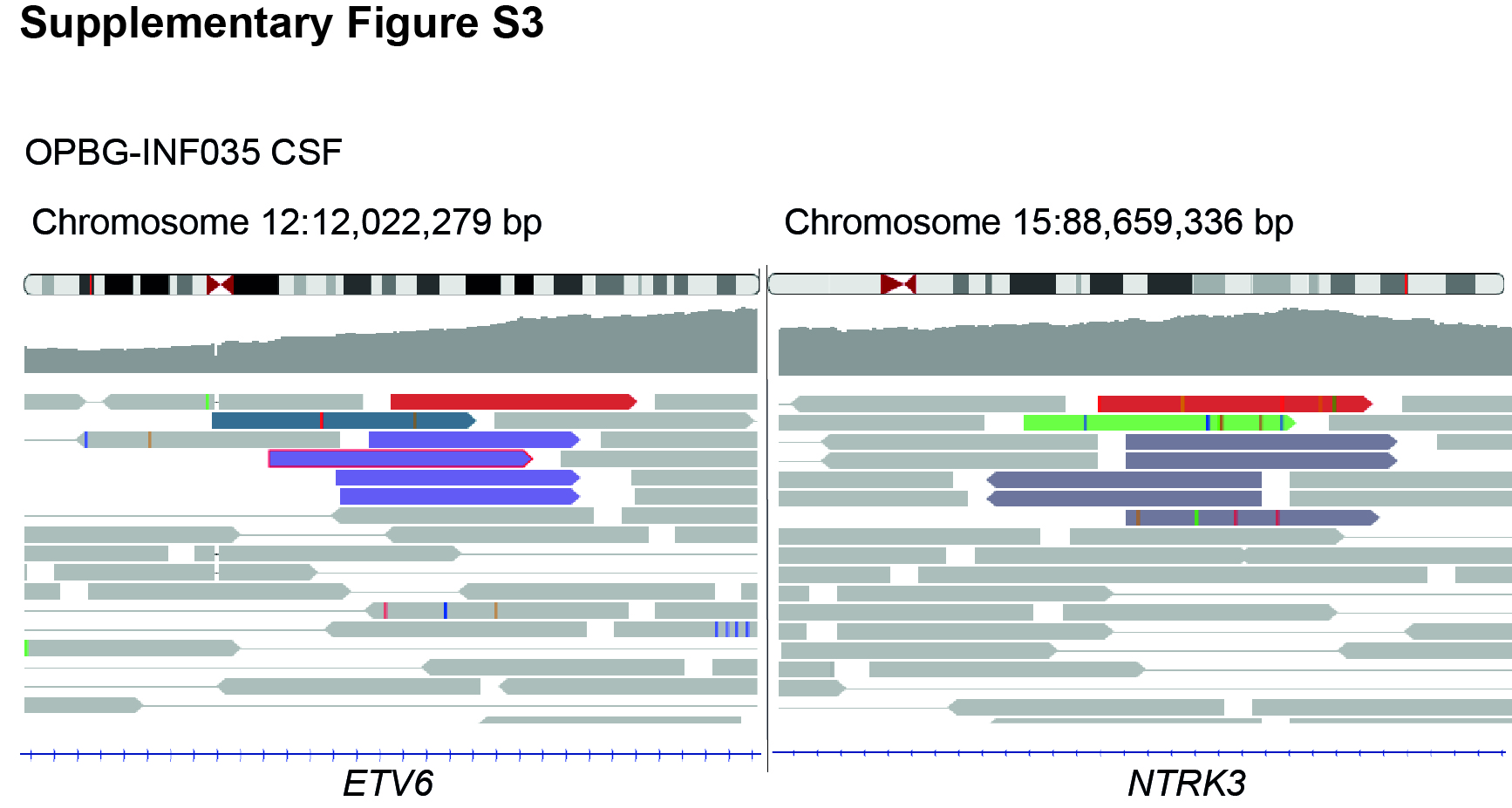
**

**Supplementary Figure S3** – *Detection of ETV6:NTRK3 fusion in CSF.* Integrative Genomics Viewer IGV) snapshot of *ETV6* and *NTRK3* detected from ctDNA-CSF (OPBG_INF_035). Reads supporting the *ETV6:NTRK3* fusion are coloured in purple/grey.

**
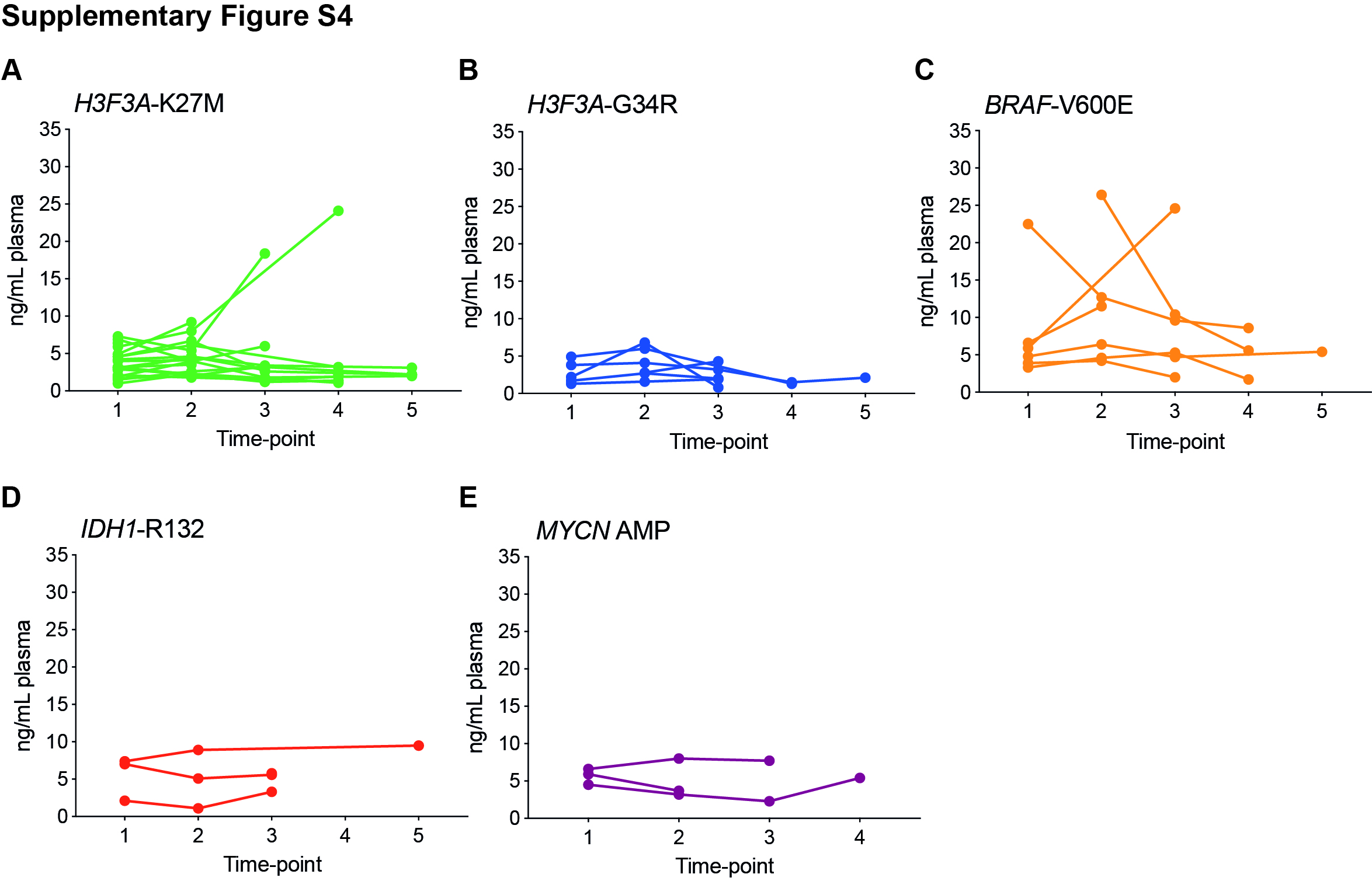
**

**Supplementary Figure S4** – *Assessment of plasma cfDNA concentration over time in the HERBY cohort.* (A) cfDNA concentrations (y axis) plotted against time-point of sampling for HERBY patients with *H3F3A*-K27M mutations. (B) cfDNA concentrations (y axis) plotted against time-point of sampling for HERBY patients with *H3F3A*-G34R mutations. (C) cfDNA concentrations (y axis) plotted against time-point of sampling for HERBY patients with *BRAF*-V600E mutations. (D) cfDNA concentrations (y axis) plotted against time-point of sampling for HERBY patients with *IDH1*-R132 mutations. (E) cfDNA concentrations (y axis) plotted against time-point of sampling for HERBY patients with *MYCN* amplification.

**SUPPLEMENTARY TABLES**

| **ddPCR assay** | **NGS VAF** | **ddPCR**  **VAF** | **Dilution** | **LoD**  **(%)** | **Mutant droplets** | **Mutant copies/ul** | **WT copies/ul** |
| --- | --- | --- | --- | --- | --- | --- | --- |
| *H3F3A-*K27M-BioRad | 81 | 79.3 | 1/100 | **0.793** | 10 | 0.33 | 53.22 |
| *H3F3A*-K27M-custom | 81 | 79.1 | 1/100 | **0.791** | 17 | 0.66 | 63.63 |
| *H3F3A*_G34R-BioRad | 48 | 50.3 | 1/1000 | **0.050** | 2 | 0.07 | 50.30 |
| *BRAF*_V600E-custom | 49 | 50.4 | 1/1000 | **0.050** | 2 | 0.07 | 65.30 |
| *IDH1*-R132S-custom | 13 | 16.5 | 1/100 | **0.165** | 2 | 0.07 | 60.60 |
| *IDH1*-R132H-custom | 40 | 43.6 | 1/1000 | **0.044** | 2 | 0.07 | 63.20 |
| *IDH1*-R132G-custom | 45 | 43.1 | 1/1000 | **0.043** | 4 | 0.13 | 72.00 |
| *TP53*-C238Y-custom | 99.3 | 100 | 1/100 | **0.993** | 19 | 0.71 | 180.61 |
| *TP53*-R282W-custom | 38.6 | 37.0 | 1/100 | **0.370** | 2 | 0.07 | 74.30 |
| *ACVR1*-R328V-custom | 54 | 64.3 | 1/100 | **0.643** | 14 | 0.52 | 181.14 |
| *PIK3CA*-E542K-custom | 5 | 4.1 | 1/100 | **0.041** | 2 | 0.07 | 145.10 |
| *PIK3CA*-H1047R-custom | 30 | 24.0 | 1/100 | **0.240** | 5 | 0.03 | 92.60 |

Supplementary Table 1 - *ddPCR assay limit of detection results.*

Samples were run in duplicate and merged data is provided. Shown are the mutations assessed by ddPCR, the biological source material, variant allele frequency (VAF), sample dilution and limit of detection (LoD), number of mutant droplets as well as number of copies of mutant and wild-type per µl.

| **Assay** | **FW** | **RV** | **WT-probe** | **Dye** | **Mutant-probe** | **Dye** |
| --- | --- | --- | --- | --- | --- | --- |
| *H3F3A*-K27M | GGTAAAGCACCCAGGAAG | CAAGAGAGACTTTGTCCC | TC+GC+A+A+GA+GT+GC | HEX | TC+GC+A+**T**+GA+GTGC | FAM |
| *BRAF*-V600E | CATGAAGACCTCACAGTAAAAATAGGTGAT | TGGGACCCACTCCATCGA | CTAGCTACAGTGAAATC | VIC | TAGCTACAGAGAAATC | FAM |
| *ACVR1-*G328V | GCTAGTGGTCTTGCACATTTGC | CTCTTTAAATCTCGATGGGCAATGG | ACCCAAGGGAAACCA | VIC | ACCCAAGTGAAACCA | FAM |
| *IDH1*-R132G | CTTGTGAGTGGATGGGTAAAACCTA | CACATTATTGCCAACATGACTTACTTGAT | AAGCATGACGACCTATG | VIC | AAGCATGACCACCTATG | FAM |
| *IDH1*-R132H | CTTGTGAGTGGATGGGTAAAACCTA | CCAACATGACTTACTTGATCCCCATA | CATCATAGGTCGTCATGC | VIC | ATCATAGGTCATCATGC | FAM |
| *IDH1-*R132S | CTTGTGAGTGGATGGGTAAAACCTA | CACATTATTGCCAACATGACTTACTTGAT | CATAAGCATGACGACCTAT | VIC | CCATAAGCATGACTACCTAT | FAM |
| *TP53-*C238Y | TGGCTCTGACTGTACCACCAT | GATGGGCCTCCGGTTCAT | ACAACTACATGTGTAACAGT | VIC | ACAACTACATGTATAACAGT | FAM |
| *TP53*-R282W | GCTTTGAGGTGCGTGTTTGTG | CTTTCTTGCGGAGATTCTCTTCCT | TGCGCCGGTCTCT | VIC | TGCGCCAGTCTCT | FAM |
| *PIK3CA*-E542K | GGGAAAATGACAAAGAACAGCTCAA | GCACTTACCTGTGACTCCATAGAAA | CCTCTCTCTGAAATCA | VIC | CCTCTCTCTAAAATCA | FAM |
| *PIK3CA*-H1047R | GCAAGAGGCTTTGGAGTATTTCATG | GCTGTTTAATTGTGTGGAAGATCCAA | CCACCATGATGTGCATC | VIC | CACCATGACGTGCATC | FAM |

**Supplementary Table S2** - *ddPCR primers.*

Sequences for assays used to detect mutations in *H3F3A* (K27M), *BRAF* (V600E), *ACVR1* (G328V), *IDH1* (R132G, R132H and R132S), *TP53* (C238Y and R282W) and *PIK3CA* (E542K and H1047R). “+” denotes locked nucleic acid bases.
